# Changes in glutamatergic neurotransmission within the migraine cycle

**DOI:** 10.1186/1129-2377-14-S1-P126

**Published:** 2013-02-21

**Authors:** G Cosentino, P Paladino, B Fierro, F Brighina

**Affiliations:** 1 University of Palermo, Italy

## Background

Although some neurophysiological studies have showed cortical excitability changes during different phases of the migraine cycle, the pathophysiological mechanisms underlying attacks recurrence remain unknown. Here we evaluated the response of the migraine motor-cortex to brief trains of 5-Hz repetitive transcranial magnetic stimulation (rTMS) in order to study, indirectly, presynaptic mechanisms of glutamatergic neurotransmission across the different phases of the migraine cycle.

## Methods

40 migraine with aura (MwA) and 40 migraine without aura (MwoA) patients underwent suprathreshold (130% of the resting motor threshold) brief trains of 5-Hz-rTMS to the motor-cortex, recording Motor Evoked Potentials (MEPs) at each train stimulus. Patients were studied whatever the phase of the migraine cycle: interictal (n=51), preictal (n=9), ictal (n=10) or postictal (n=10).

## Results

As we previously showed [1], in the interictal phase MEPs decreased significantly in size during 5-Hz trains. A significant greater inhibitory response was recorded during the ictal and post-ictal phase. Conversely, in the pre-ictal phase, we observed a facilitatory response to the trains similar to that of normal subjects. No significant differences were recorded between MwA and MwoA patients.

## Conclusions

Our results support the hypothesis that in migraine a transient increase in intracortical glutamatergic activity could trigger the migraine attack. Inhibitory homeostatic mechanisms of glutamate release could be involved in the resolution of the migraine attack and in preventing further attacks.
